# The 2011 Medical Molecular Hydrogen Symposium: An inaugural symposium of the journal *Medical Gas Research*

**DOI:** 10.1186/2045-9912-1-10

**Published:** 2011-06-07

**Authors:** Shigeo Ohta, Atsunori Nakao, Kinji Ohno

**Affiliations:** 1 Department of Biochemistry and Cell Biology, Institute of Development and Aging Sciences, Graduate School of Medicine, Nippon Medical School, 1-396 Kosugi-machi, Nakahara-ku, Kawasaki-city, Kanagawa 211-8533, Japan; 2 Thomas E Starzl Transplantation Institute, Department of Surgery, University of Pittsburgh Medical Center, 200 Lothrop Street, Pittsburgh, PA 15213, USA; 3 Division of Neurogenetics, Center for Neurological Diseases and Cancer, Nagoya University Graduate School of Medicine, 65 Tsurumai, Showa-ku, Nagoya 466-8550, Japan

## Abstract

This report summarizes a brief description/history of the Hydrogen Research Meetings as well as key presentations/oral abstracts delivered in the most recent symposium. Additionally, we introduced 38 diseases and physiological states for which hydrogen exhibits beneficial effects.

## Introduction

**Figure 1:**
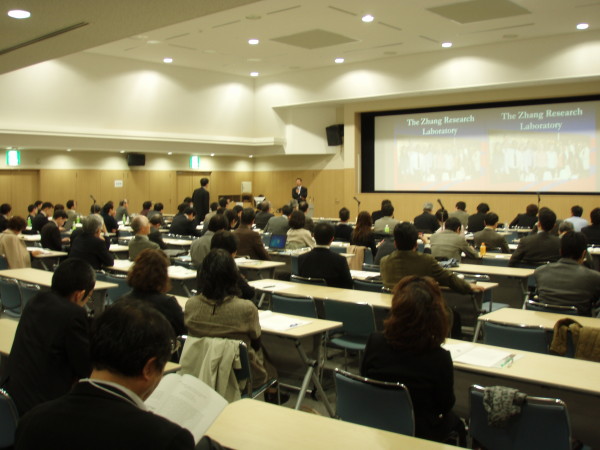
**A snap shot of the Medical Molecular Hydrogen Symposium in 2011**.

Novel medical gases are expected to provide us with more effective therapeutic interventions and preventive medicine. In the past decades, there has been extraordinary, rapid growth in our knowledge of gaseous molecules, including nitric oxide, carbon monoxide, and hydrogen sulfide, which have been known to play important roles in biological systems. Additionally, since Dr. Shigeo Ohta's group's pioneering paper was published in the June 2007 *Nature Medicine *showing the potency of hydrogen as a therapeutic gas for oxidative stress-mediated diseases including cerebral infarction [1], basic and clinical hydrogen research has resurfaced. In Japan, the birthplace of hydrogen gas research, Dr. Ohta (Nippon Medical School), who is currently serving as an Associate Editor of *Medical Gas Research*, organized annual "Medical Molecular Hydrogen Research Meetings" in 2009 and 2010 to provide investigators with focused opportunities to share their rapid scientific progress. Most recently, we organized the Medical Molecular Hydrogen Symposium on February 18-19, 2011 at the Nagoya University Hall (Figure 1). The latest meeting is a "kick-off" inaugural meeting for the newly launched journal *Medical Gas Research *(MGR), which aims to promote the exchange and dissemination of the latest scientific findings.

This report summarizes a brief description/history of the Hydrogen Research Meetings as well as key presentations/oral abstracts delivered in the most recent symposium.

## First Medical Molecular Hydrogen Research Meeting, 2009

The first scientific meeting organized by Dr. Ohta was held on February 7, 2009 in Tokyo. 42 scientists and clinicians from 30 individual institutes were invited. The aim of the meeting was to unite innovative investigators to discuss and propagate medical hydrogen research. Dr. Ohta delivered the keynote presentation, in which he gave a brief history of hydrogen medicine and emphasized the huge impact of his report published in *Nature Medicine*. He pointed out the great interest in the field, expressed in more than 30 personal communications with investigators, and the resulting need to widen the scope of basic/clinical research to the whole world. He mentioned the successful application of hydrogen gas in a rat neonatal hypoxic brain injury model in collaboration with Dr. Xuejun Sun (Second Military Medical School, Shanghai, China) [2], who is currently serving as an Associate Editor. Dr. Atsunori Nakao (Department of Surgery, University of Pittsburgh), who is also an Associate Editor, presented the promising preliminary results of a collaborative study with Dr. Ohta's group in which hydrogen water was applied in a rat kidney transplant model. Dr. Nakao's report clearly showed survival benefits for transplant recipients. He received a research award at this meeting and his report was later published in *Kidney International *[3,4]. Dr. Takahisa Kawai (Forsythe Research Institute, Boston, MA, USA), who is an editorial board member, focused on hydrogen generated by intestinal bacteria. His initial studies elegantly demonstrated the critical physiological roles of gut microflora-derived hydrogen [5]. There was a general consensus that both clinicians and researchers in the field of molecular hydrogen research should gather and exchange accumulating knowledge in future annual meetings.

## Second Medical Molecular Hydrogen Research Meeting, 2010

The second meeting was also organized by Dr. Ohta on February 3, 2010 in Tokyo. 47 basic scientists and clinical physicians, as well as 23 corporate participants were invited and shared the latest developments in medical issues related to hydrogen. This meeting hosted a keynote lecture, an invited lecture, two special lectures, and twelve platform presentations. After Dr. Ohta began his keynote lecture by remarking on the impressive progress over the last year, Dr. Sun gave an invited lecture and introduced the great effects of intraperitoneal administration of saline dissolved with H_2 _in several model animals. Dr. Takashi Asada (Department of Psychiatry, Tsukuba University), an authority on Alzheimer disease, presented the results of clinical studies involving patients with mild cognitive impairment (MCI). He started clinical intervention studies on MCI patients by orally administering hydrogen water; the project is still in progress. Dr. Toru Yoshikawa (Kaohsiung Medical University, Taiwan) also gave a special lecture on the physical aspects of hydrogen effects. He presented the physical characteristics of molecular hydrogen's interaction with water in biological systems.

## Third Medical Molecular Hydrogen Research Meeting, 2011

The third meeting was organized by Dr. Kenji Ohno (Nagoya University Graduate School of Medicine), an editorial board member, and held in Nagoya on Feb 18-19, 2011. This symposium mainly focused on molecular hydrogen and covered a wide-range of therapeutic gases, including hydrogen sulfide (H_2_S), nitric oxide (NO), and carbon monoxide (CO). A total of 98 academic and 53 corporate registrants attended the meeting from Japan (144), USA (5), Korea (1), and Taiwan (1). The meeting's timetable, titles, and speakers are summarized in Table 1. We also introduced 38 diseases and physiological states for which hydrogen exhibits beneficial effects (Table 2).

**Table 1:** Scientific Program of the Medical Molecular Hydrogen Symposium in 2011

Feb. 18, 2011	Title	Speaker
13:00	**Opening Remarks**	Kinji Ohno, Nagoya Univ.
13:05	**Keynote Lecture **"Recent progress towards hydrogen medicine"	Shigeo Ohta, Nippon Medical Sch.
13:35	**Scientific Session I**	
	1. Effects of hydrogen-rich water on kidney functions in SHR.Cg-*Lepr^cp^*/NDmcr rat - a metabolic syndrome model rat	Michio Hashimoto, Shimane Univ.
	2. Hydrogen-rich UW solution attenuates renal cold ischemia reperfusion injury	Toyofumi Abe, Osaka Univ.
	3. Therapeutic effect of maternal hydrogen water administration in a rat model of fetal brain damage	Yukio Mano, Nagoya Univ.
	4. Effects of hydrogen water on human skin and hair	Yoshiaki Kurita, Hosei Univ.
14:23	Coffee Break	
14:50	**Scientific Session II**	
	5. Consumption of hydrogen water prevents memory impairment accompanying neurodegeneration in transgenic mice	Ikuroh Ohsawa, Metropolitan Instittue of Gerontology
	6. Appearance of hydrogen gas in the human skin after the ingestion of hydrogen-rich water and inhalation of hydrogen gas	Kazutoshi Nose, National Cerebral and Cardiovascular Center
	7. Effectiveness of long-term intake of hydrogen rich water in a patient with MELAS	Mikio Hirayama, Kasugai Municipal Hospital
	8. Effectiveness of hydrogen rich water on myopathy	Tohru Ibi, Aichi Medical Univ.
	9. Inhaled hydrogen gas therapy for prevention of lung transplant-induced ischemia/reperfusion injury in rats	Tomohiro Kawamura, Univ. of Pittsburgh
15:50	**Invited Lecture I **"Deadly gas can save a life! -Preclinical studies using carbon monoxide/hydrogen-"	Atsunori Nakao, Univ. of Pittsburgh
16:30	Break	
16:40	**Invited Lecture II **"Medical Gas Research"	John Zhang, Loma Linda Univ.
17:20	**Invited Lecture III **"A hypothesis on biological protection from space radiation through the use of therapeutic gases"	Michael P. Schoenfeld, NASA Marshall Space Flight Center
18:10	Banquet	

Feb. 19, 2011		

9:30	**Symposium I **"CO as a therapeutic modality"	Organizer: Atsunori Nakao, Univ. of Pittsburgh
	S1-1. Is exhaled carbon monoxide a marker of oxidative stress?	Tohru Takahashi, Okayama Prefecture Univ.
	S1-2. A tracer analysis study demonstrates redistribution of endogenous carbon monoxide from blood to tissue in human body	Makoto Sawano, Saitama Medical Univ.
	S1-3. Translational research of carbon monoxide therapy using miniature swine	Hisashi Sahara, Kagoshima Univ.
10:30	**Special Lecture **"Function of hydrogen sulfide and its therapeutic applications"	Hideo Kimura, National Center of Neurology and Psychiatry
11:10	Break	
11:20	**Scientific Session III**	
	10. Hydrogen from intestinal bacteria is protective for Con A-induced Hepatitis	Mikihito Kajiya, The Forsyth Institute
	11. Abnormal breath hydrogen production by ingestion of lactulose in Parkinson's disease	Masaaki Hirayama, Nagoya Univ.
	12. A new portable breath hydrogen analyzer and its clinical application	Akito Shimouchi, National Cerebral and Cardiovascular Center
	13. Application of hydrogen water in the dental field	Noriyuki Tanaka, Uchida Dental Clinic
12:08	Lunch	
12:45	**Symposium II **"Biological Effects of NO"	Hirosuke Kobayashi, Kitasato Univ.
	S2-1. NO-ROS cellular signaling mediated via nitrated cyclic nucleotide	Hideshi Ihara, Osaka Prefecture Univ.
	S2-2. Relationship between protein S-nitrosylation and neuronal death	Takashi Uehara, Wakayama Mediccal Univ.
	S2-3. Role of nitrosative stress in chronic obstructive pulmonary disease	Ryujiro Sugimoto, Iwakuni Clinical Center
	S2-4. Effects of simultaneous inhalation of nitric oxide and hydrogen on mouse myocardial ischemia-reperfusion injury	Hirosuke Kobayashi, Kitasato Univ.
14:15	**Scientific Session 4**	
	14. Effect of hydrogen rich water against a progression of disease and a formation of liver tumor in NASH model mouse	Daisuke Kawai, Okayama Univ.
	15. The dynamic movement of H2 in a liver and its effects	Naomi Kamimura, Nippon Medical Univ.
	16. Molecular hydrogen effectively protects cereulide-induced liver injury by suppressing apoptosis	Sayaka Sobue, Chubu Univ.
14:51	**Closing Remarks**	Kinji Ohno, Nagoya Univ.

**Table 2:** Thirty-eight diseases and physiological states for which hydrogen effects are reported

Disease/Physiology	Species	Source of H_2_	Reference
**Brain**			
Cerebral infarction	rodent	gas	[1]
Superoxide in brain	rodent	water	[6]
Neonatal brain hypoxia	rodent	gas	[2,7]
	rodent	saline	[8]
	pig	gas	[9]
Restraint-induced dementia	rodent	water	[10]
Alzheimer's disease	rodent	saline	[11]
Senile dementia	rodent	water	[12]
Parkinson's disease	rodent	water	[13,14]
Hemorrhagic cerebral infarction	rodent	gas	[15]
Traumatic brain injury	rodent	gas	[16]
**Spinal cord**			
Spinal cord injury	rodent	saline	[17]
**Eye**			
Glaucoma	rodent	eye drop	[18]
Corneal alkali-burn	rodent	eye drop	[19]
**Ear**			
Hearing disturbance	rodent	medium	[20]
	rodent	gas	[21]
	rodent	water	[22]
**Lung**			
Lung cancer	Cells	medium	[23]
Oxygen-induced lung injury	rodent	saline	[24,25]
Lung transplantation	rodent	gas	[26]
**Heart**			
Myocardial infarction	rodent	gas	[27]
	rodent	saline	[28]
Heart transplantation	rodent	gas	[29]
Irradiation-induced heart injury	rodent	water	[30]
**Liver**			
Hepatic ischemia	rodent	gas	[31]
Hepatitis	rodent	bacteria	[5]
Obstructive jaundice	rodent	saline	[32]
**Kidney**			
Cisplatin nephropathy	rodent	gas, water	[33]
	rodent	water	[34]
Hemodialysis	human	dialysis	[35,36]
Kidney transplantation	rodent	water	[4]
**Pancreas**			
Acute pancreatitis	rodent	saline	[37]
**Intestine**			
Intestinal graft	rodent	gas	[3]
	rodent	saline	[38,39]
Ulcerative colitis	rodent	gas	[40]
**Blood vessel**			
Atherosclerosis	rodent	water	[41]
**Metabolism**			
Diabetes mellitus type 2	human	water	[42]
Metabolic syndrome	human	water	[43]
Obesity/Diabetes	rodent	water	[44]
**Cancer**			
Tongue carcinoma	cells	medium	[45]
**Inflammation and allergy**			
Allergy type I	rodent	water	[46]
Sepsis	rodent	gas	[47]
Zymosan-induced inflammation	rodent	gas	[47]
**Others**			
Multipotent stromal cells	cells	gas	[48]
Radiation injury	cells	medium	[49,50]

Although the observations are not directly relevant to diseases, Turmeric [51] and acarbose [52] increase hydrogen production by intestinal bacteria in humans.

### Hydrogen

Dr. Ohta gave a keynote lecture and introduced a number of hydrogen's potent efficacies on a broad spectrum of diseases in animal and human models, as well as the emerging molecular bases of hydrogen's effects. He emphasized the following points: (i) In the three and a half years since the first hydrogen paper was published in *Nature Medicine*, more than 70 original papers have been published in leading biological/medical journals. Based on cumulative knowledge, beneficial biological effects of hydrogen have been established with no doubt. (ii) There are several ways to intake or consume hydrogen, including inhaling hydrogen gas, drinking water dissolved with hydrogen (hydrogen water), taking a hydrogen bath, injecting hydrogen saline, dropping hydrogen saline into the eye, and increasing production of intestinal hydrogen by bacteria. (iii) Hydrogen shows not only anti-oxidative stress effects, but also has various anti-inflammatory and anti-allergic effects. (iv) The primary molecular target of hydrogen remains unknown. In their first report published in 2007 [1], Dr. Ohta's group indicated that cells cultured in H_2_-rich medium were protected against oxidative stress by the hydroxyl radical-scavenging activity of H_2_; however, recent evidence clearly shows that the scavenging property is not the only explanation for the potent beneficial effects of hydrogen. For example, the amount of orally administered H_2 _may not be enough to scavenge hydroxyl radicals. In addition, it is likely that the dwell time of H_2 _in the body is too short to scavenge a large amount of hydroxyl radicals that are continuously generated. (v) Several reports demonstrate an effect on the regulation of gene expressions and protein-phosphorylations; however, the transcriptional factors and kinases involved in the functions afforded by H_2 _have not been identified. It also remains unknown whether the regulations are directly performed by H_2_. (vi) The amount of administered H_2 _is independent of the extent of effects. Intestinal bacteria seem to produce more than 1 liter of H_2 _gas per day, whereas the amount of H_2 _originating from drinking hydrogen water is less than 50 ml. Nevertheless, additional H_2 _in drinking hydrogen water is unambiguously effective. Many mysteries of hydrogen therapy remain unsolved. He closed his talk by emphasizing that the molecular mechanisms underlying the amazing effects of a very small amount of H_2 _remain elusive.

Sixteen platform speakers presented clinical and basic aspects of the medical application of molecular hydrogen. Among them were three treatments for patients with neurological and dental diseases. Dr. Mikio Hirayama (Department of Neurology, Kasugai City Hospital) and his colleagues presented their clinical report of treatment for a patient with mitochondrial encephalomyopathy, lactic acidosis, and stroke-like episode (MELAS) syndrome. He reported that a 33-year-old female patient was successfully treated by drinking hydrogen-containing water for one and half years, which reduced the frequency of episodic cerebral ischemia. Dr. Tohru Ibi (Department of Neurology, Aichi Medical School) conducted an open label trial on mitochondrial disorders and inflammatory myopathies, and demonstrated a remarkable reduction of several serum markers specific for myelopathy. He also conducted a double-blind crossover trial; however, the trial showed no significant effects, which was likely due to a small amount of hydrogen water and to a short observation period. Both reports suggest that oral administration of hydrogen water is likely to be effective for mitochondrial diseases. Dr. Noriyuki Tanaka (Uchida Dental Clinic) reported that direct dental application of hydrogen water on injured regions reduced inflammation and promoted healing in dental operations, including tooth extraction.

*Medical Gas Research*'s editor-in-chief Dr. John Zhang (Department of Neurosurgery, Loma Linda University, CA) gave a greeting talk, introducing the aims and scope of the journal. Dr. Zhang pointed out the importance of stimulating medical gas research and collaborating with a wide-range of people in various fields.

Dr. Michael P. Schoenfeld from NASA gave a special lecture on the potential application of H_2 _to protect astronauts from radiation-mediated injury during long space travel. Cosmic radiation induces serious oxidative stress, which is one of the major issues to be resolved by hydrogen research.

### Other Medical Gases (NO, CO, and H_2_S)

Mammals produce NO, CO, and H_2_S by their native enzymes; however, mammals lack an enzyme to produce H_2_. All four gases modulate signaling pathways and have some therapeutic effects. Thus, we invited leading NO, CO, and H_2_S researchers to the symposium.

Dr. Hideo Kimura (National Center of Neurology and Psychiatry), an editorial board member, gave a special lecture on hydrogen sulfide (H_2_S). Dr. Kimura discovered biological roles for H_2_S. He reviewed studies on H_2_S from past to present and emphasized the potential for actual medical applications.

Dr. Nakao organized a mini-symposium on carbon monoxide (CO). In the mini-symposium, the discrepancy between the animal and human models was discussed. CO binds to human hemoglobin more strongly than to murine hemoglobin; this seems to be the reason why CO is more toxic in clinical studies.

Dr. Hirosuke Kobayashi (Department of Respiratory Medicine, Kitasato University), an editorial board member, organized a mini-symposium on nitric oxide (NO). Dr. Kobayashi explained the contradictory properties of NO; while it is medically beneficial, it is also toxic and enhances oxidative stress. NO has been approved as a therapeutic agent in clinical practice. He presented the amazing effects of inhaling an NO and H_2 _mixture. NO enhances oxidative stress and induces production of peroxynitrite, and H_2 _reduces peroxynitrite derived from NO. Indeed, he presented that the mixture of NO and H_2 _improves the number of surviving myocytes in a rodent model of myocardial infarction. The mixture of NO and H_2 _may be a promising modality for clinical applications.

In addition to NO, CO, and H_2_S, Dr. Zhang addressed potential effects of inhaled xeon and helium in animal models in his invited lecture.

### Commercial products

Six companies financially supported the meeting by demonstrating their new commercial products. Melodian Co Ltd. presented a sports drink containing H_2_, and Miz Co Ltd. introduced an interesting device to prepare a clinically applicable H_2_-rich infusion solution by simply soaking an infusion bag in hydrogen water. With this method, no other treatment is necessary to prepare hydrogen infusion solution without opening the infusion bag. Taiyo Co Ltd. presented reasonably priced devices to measure H_2 _gas in breath. Takaoka Co Ltd. demonstrated a tiny home apparatus to produce hydrogen water. Doctor's Choice Co Ltd. demonstrated powders to prepare a hydrogen bath at home using a new material that releases H_2 _gas. These new products will help expand our knowledge and applicability of hydrogen research.
